# Community size and perception of older adults in the Cook Islands

**DOI:** 10.1371/journal.pone.0219760

**Published:** 2019-07-17

**Authors:** Tomasz Frackowiak, Anna Oleszkiewicz, Corinna E. Löckenhoff, Agnieszka Sorokowska, Piotr Sorokowski

**Affiliations:** 1 Institute of Psychology, University of Wroclaw, Wroclaw, Poland; 2 Interdisciplinary Center “Smell & Taste”, Department of Otorhinolaryngology, TU Dresden, Dresden, Germany; 3 Department of Human Development, Cornell University, Ithaca, NY, United States of America; 4 Department of Psychotherapy and Psychosomatic Medicine, TU Dresden, Dresden, Germany; University of Bologna, ITALY

## Abstract

Attitudes towards aging are often negative, a phenomenon known as ageism. However, personal contact with older adults and intergenerational exchange in the context of close families may mitigate such negative tendencies. So far, these effects have been studied in Western and industrialized contexts. The present study extended this work to the Cook Islands archipelago, a group of islands in the South Pacific characterized by low levels of industrialization and relative isolation from foreign influences. We tested the hypothesis that attitudes toward aging in the Cook Islands would be more positive than in the world at large, and that, within the archipelago, attitudes towards aging would be more positive in smaller, less industrialized communities with closer family ties. Participants (n = 70), were recruited from three islands varying in community size and strength of the family ties among inhabitants. They rated their aging attitudes on four dimensions. Contrary to our hypotheses, attitudes in the Cook Islands did not differ from those reported in industrialized nations and did not vary significantly across islands, even after controlling for personal contact to older adults. Potential limitations and implications for future research are discussed.

## Introduction

Human population aging was first recognized as a serious demographic challenge over sixty years ago [1, 2], but it was primarily discussed as a problem of industrialized countries. In recent years, developing countries are beginning to face this challenge as well [3] necessitating a better understanding of the factors that govern the perception and treatment of older adults across the world [4–6]. Of particular interest are the role of community characteristics and family ties that may in turn affect access to potential caregivers [7, 8].

Unfortunately, interactions with older adults may be negatively affected by *ageism* [9, 10], a tendency to perceive and treat individuals more negatively because of their age [10, 11]. Like other stereotypes, ageism can be mitigated by enhanced personal contact with subjects of prejudice, a phenomenon known as the *contact hypothesis* [12]. Personal contact with old people decreases negative attitudes [13] through more realistic perceptions and reduced anxiety [14–16]. This effect is especially visible in children interacting with older people [8, 13].

While the contact hypothesis operates at the individual level, societal factors may influence attitudes towards aging as well. Specifically, *modernization theory* [17, 18] has argued that increasing industrialization decreases the societal status of older people because it disrupts traditional extended families and decreases the value of older adults’ experience-based knowledge. In support of this idea, it has been shown that in traditional societies, perception of elderly people are more positive due to the respect and honor given to seniors [19–22]. Beyond modernization, perception of elderly people in less industrialized societies may also be linked to community size. Members of small communities usually know each other, and–according to the contact hypothesis—this personal contact may mitigate negative age stereotypes.

So far, such effects have mostly been studied in Western and industrialized contexts (for a meta-analysis see North, Fiske [23]) with only a few studies comparing traditional and industrialized societies (e.g., [22]). The present study extends this work to the Cook Islands archipelago, a group of islands in the South Pacific characterized by low levels of industrialization and relative isolation from foreign markets [24, 25].

We gathered data from three islands varying in community size, contact to the outside world, and exposure to mainstream media. We performed both *cross-cultural comparisons*, testing the hypothesis that attitudes toward aging in the Cook Islands would be more positive than in industrialized countries around the world and *within-culture comparisons*, testing the hypothesis that—within the archipelago—attitudes towards aging would be more positive in smaller, less industrialized communities characterized by closer family ties, greater personal acquaintance with older adults, and lower exposure to mainstream media. Following prior research (e.g., [23, 26]) the latter analyses also controlled for contact to older adults.

## Materials and methods

### Participants

Participants consisted of 70 inhabitants of three islands of the Cook Islands archipelago: Rarotonga, Aitutaki, and Palmerston (for participant characteristics, see Table 1).

**Table 1:** Sample characteristics and aging attitudes by location.

	Rarotonga	Aitutaki	Palmerston	Total	p_Diff_	BF_01_
N	39	16	15	70		
% Female	64	75	47	63	.26	2.42
Age	32.62 (11.82)	31.19 (10.30)	26.87 (15.72)	31.06 (12.46)	.32	3.18
Older acquaintances	103.92 (70.53)	49.88 (49.88)	13.93 (17.56)	72.29 (69.03)	.00	3.12*10^−4^
**Aging attitudes**						
Daily tasks	-0.28 (0.86)	-0.38 (0.62)	0.00 (0.76)	-0.24 (0.79)	.38	3.77
Satisfaction	-0.23 (0.84)	-0.38 (0.81)	0.27 (0.7)	-0.16 (0.83)	.07	1.04
Authority	0.31 (0.77)	0.5 (0.73)	0.2 (0.77)	0.33 (0.76)	.53	4.81
Wisdom	0.44 (0.64)	0.31 (0.6)	0.47 (0.64)	0.41 (0.63)	.76	6.13

p_Diff_ = p-value testing for significant differences across islands, BF_01_ = Bayes Factor in support of the null Hypothesis; standard deviations for continuous measures are shown in parentheses

*Rarotonga*, with 13,000 inhabitants, is the biggest island of the Cook Islands where Avarua, the capital, is located. It is the major tourist destination within the archipelago and home of the international airport as well as numerous hotels and other infrastructure [25]. The study was conducted in Avarua at the Sunday market. North from Rarotonga lies *Aitutaki*, an island with a population of approximately 2,000 people. Aitutaki is the second most populated island of the Cook Islands. The study was conducted in the main village of Aitutaki—Arutanga. Finally, *Palmerston* is a tiny atoll located 500 km north-west from Rarotonga and is inhabited by approximately 60 people who rarely have contact with tourists or other inhabitants of the Cook Islands archipelago, thus it meets the requirement of a small, isolated community. The present inhabitants of Palmerston comprise three families of the same surname: Masters. There are few scientific works documenting the history of Palmerston, but some additional information can be found in Pryor [27] and Hendery [28].

Table 1 shows demographic characteristics for each sample. All data are available at https://figshare.com/articles/cook_islands_PLOS_xlsx/8256503. As seen in the last column of the table, the samples from the different islands had similar gender and age distributions. However, the number of older acquaintances varied across islands such that people living in smaller communities reported knowing fewer older adults.

### Procedure

The study was approved by Cooks Islands National Research Committee, the ethical board of the Institute of Psychology at the University of Wroclaw, and Cornell University Institutional Review Board. Data was collected during individual meetings (after obtaining informed consent from the respondents) conducted by the researchers in English (official language at the Cook Islands, commonly known by the inhabitants). Questions were adapted from Sorokowski et al. [22] and Löckenhoff et al. [26] and assessed aging attitudes with regard to life satisfaction, performance of daily tasks, family authority, and wisdom. For each of these characteristics, participants were asked to indicate whether life satisfaction, performance of daily tasks, family authority and wisdom increased (coded as 1 for the purpose of further statistical analyses), remained stable (coded as 0) or decreased (coded as -1) in older adults. Lower scores indicate perceptions of age-related decrements in functioning or status and thus more negative aging attitude. Participants also reported how many older people they knew personally and researchers recorded the respondents’ age and gender.

## Results

Initial analyses examined the general direction of aging attitudes in our sample. We performed a repeated-measures general linear model (GLM) with four aspects of aging included as a within subject factors (life satisfaction vs performance of daily tasks vs family authority vs wisdom). We found that aging attitudes differed across characteristics, F(3, 207) = 13.77, p < .001, partial eta-squared = .17. Within-subjects contrasts indicated that, consistent with the prior literature [26], Cook islanders reported more negative attitudes for age-related changes in daily tasks and life satisfaction than for changes in family authority and wisdom (all *p*s < .001). Given the low sample size, we conducted an analogous, additional Bayesian analysis which confirmed that the alternative hypothesis (i.e., there are differences between four aspects of aging attitudes) is far more likely than the null hypothesis, BF_10_ = 2.498*10^6^. Negative attitudes for age-related changes in life satisfaction were far more likely to be observed as compared to authority (BF_10_ = 70.9) and wisdom (BF_10_ = 753.8). Further, negative attitudes for age-related changes in performance were more likely to be observed as compared to changes in authority (BF_10_ = 171.9) and wisdom (BF_10_ = 12513.2).

For the purpose of cross-cultural comparisons, the aging attitude scores for the combined Cook Island sample were compared to the average scores derived from 26 industrialized cultures across the world ([26]; for this comparison, the original 5-point scores were transformed to a 3-point scale). One-sample t-Tests (Bonferroni corrected) indicated that Cook Islanders did not differ in aging attitudes relative to industrialized societies for daily tasks (-0.38 in Löckenhoff et al. [26] vs. -0.24 in the present sample; *t*(69) = 1.46, *p* = .15), life satisfaction (-0.03 vs. -0.16; *t*(69) = 1.29, *p* = .2), family authority (0.21 vs. 0.33; *t*(69) = -1.31, *p* = .19), and wisdom (0.52 vs. 0.41; *t*(69) = 1.41, *p* = .16). The aforementioned statistics indicate a lack of discrepancies between previously published cross-cultural data and currently presented evidence from the Cook Islands.

For within-culture comparisons, we computed further GLMs with island as the independent variable, and the aging attitude scores for each of the four aspects of aging as dependent variables. Again, all comparisons between the islands were further verified with the use of Bayesian analyses. As seen in Table 1, there was a trend indicating that attitudes about life satisfaction in Palmerston were more positive than in the two other communities, F(2, 70) = 2.82, partial eta-squared = .08, *p* = .07 (BF_01_ = 1.04; inconclusive result). However, this trend was no longer visible after controlling for differences in the number of older acquaintances across islands, F(2, 70) = 1.85, partial eta-squared = .05, *p* = .17 (BF_01_ = 1.64; inconclusive result). Older people were perceived similarly across the three Islands with respect to authority, wisdom and daily task (all *p*s > .38; all BF_01_ >3.77; a lack of difference was at least 3.77 more likely than differences between islands).

## Discussion

The present study adds to the literature by providing a better understanding of aging attitudes in the Cook Islands, a low-industrialized and relatively isolated society. To the best of our knowledge, the present data are the first to provide quantitative data on views of aging within this community. In general, aging attitudes in the Cook Island archipelago were very similar to those observed in more industrialized societies across the world. Consistent with prior findings [26], Cook Islanders had more negative aging attitudes about changes in everyday tasks and life satisfaction than about changes in family authority and wisdom. Further, the numerical scores for each aspect of age-related change that we observed in the Cook Islands did not differ significantly from those observed in 26 industrialized countries [26]. Thus, the present data add to existing evidence suggesting that some aspects of aging attitudes are fairly universal. Data did not support our prediction that aging attitudes would be more positive within this developing and fairly isolated community.

We also examined variations in aging attitudes across islands. Only one of the characteristics–life satisfaction–showed a trend in the predicted direction such that residents from the small and isolated Palmerston tended to have more positive views of life-satisfaction in later life than inhabitants of the two larger islands. However, after controlling for variations in contact to older adults, this trend was no longer visible.

Although the present data allow a first glimpse of aging attitudes in the Cook Islands, further research is needed to address a variety of limitations and open questions. First, it needs to be noted that the assumptions of the current study which required our hypotheses to be tested in a small, isolated community made it very difficult to recruit large groups of participants. Our exploratory study employed a relatively modest sample, and future studies, especially those focused on complex hypotheses, should recruit larger sample sizes from a wider range of islands within the archipelago. This would allow researchers to systematically disentangle the effects of community size, contact with older adults, and relative geographic isolation. In particular, the contact hypothesis [12] which proposes that personal acquaintance with older adults is associated with more positive aging attitudes would require a more refined assessment within the Cook Island setting. Inhabitants of the Cook Islands are closely related, especially in Palmerston, where all islanders have common ancestors [27]. Members of such close-knit communities may know a smaller number of older adults, but the contacts with the elders they know are more likely to be intense and personal resulting, in an in-group positive bias [29, 30]. Thus future studies should not only assess how many older adults are known, but also capture the frequency and emotional valence of such contacts. Further research is also needed to explore the potential role of media exposure and track the type of media (e.g., Internet, TV, newsprint) and the frequency with which they are consumed to explore their relative influence on aging attitudes. Finally, future studies should expand the aspects of aging attitudes that are being considered and employ a range of assessment approaches. To allow for comparisons with prior work [22, 26] the present study focused on aging expectations for a limited set of relevant domains. Future work should extend this scope to include emotional responses as well as behavioral intentions towards the elderly and supplement quantitative approaches with qualitative accounts.
